# Applications of 3D Bioprinting in Tissue Engineering and Regenerative Medicine

**DOI:** 10.3390/jcm10214966

**Published:** 2021-10-26

**Authors:** Gia Saini, Nicole Segaran, Joseph L. Mayer, Aman Saini, Hassan Albadawi, Rahmi Oklu

**Affiliations:** 1 Division of Vascular and Interventional Radiology, Laboratory for Patient Inspired Engineering, Mayo Clinic, Phoenix, AZ 85054, USA; saini.gia@mayo.edu (G.S.); nsegaran2@gmail.com (N.S.); asaini@umkc.edu (A.S.); albadawi.hassan@mayo.edu (H.A.); 2 3D Innovations Laboratory, Support Services Building, Mayo Clinic, Phoenix, AZ 85054, USA; mayer.joseph@mayo.edu

**Keywords:** regenerative medicine, tissue engineering, 3D bioprinting, bioinks, bioreactors

## Abstract

Regenerative medicine is an emerging field that centers on the restoration and regeneration of functional components of damaged tissue. Tissue engineering is an application of regenerative medicine and seeks to create functional tissue components and whole organs. Using 3D printing technologies, native tissue mimics can be created utilizing biomaterials and living cells. Recently, regenerative medicine has begun to employ 3D bioprinting methods to create highly specialized tissue models to improve upon conventional tissue engineering methods. Here, we review the use of 3D bioprinting in the advancement of tissue engineering by describing the process of 3D bioprinting and its advantages over other tissue engineering methods. Materials and techniques in bioprinting are also reviewed, in addition to future clinical applications, challenges, and future directions of the field.

## 1. Tissue Engineering and Regenerative Medicine

**Figure 1:**
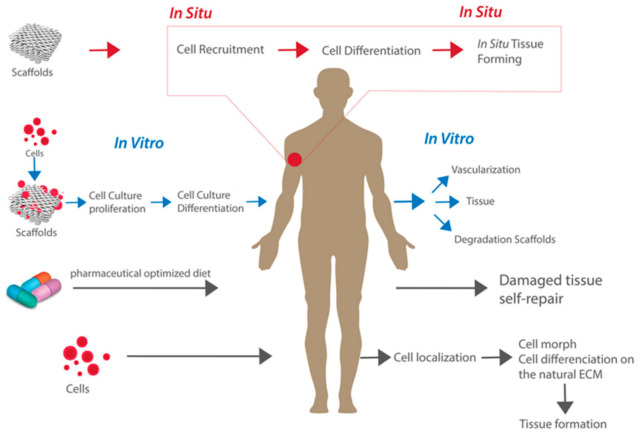
The classical approaches to tissue engineering. Reproduced under open access from [4], published by MDPI, 2019.

The ability to regenerate tissue has become increasingly more important as a novel method to restore the functional components of damaged tissues and organs [1]. Tissue engineering is an application of regenerative medicine that aims to use in vitro and in situ methods to regenerate specific tissues and restore normal biological functionality [2,3]. The classical approaches to tissue engineering include the implantation of (a) scaffolds alone, (b) isolated cells and other bioactive molecules, or (c) a combination of cells implanted within or on scaffolds to model the body’s natural extracellular matrix (ECM) and promote tissue engineering [2,3,4]. These approaches are displayed in Figure 1. There are different advantages and potential uses of each approach. On the one hand, the combination approach of cells seeded onto scaffolds tends to be the most widely used approach due to its ability to culture cells and observe the maturation process outside of the body, followed by implanting this cell-seeded 3D structural support within the body [4]. On the other hand, the implantation of a scaffold alone can provide structural support while also promoting natural cell recruitment to the area in situ. The field of regenerative medicine focuses on providing support to the body’s own self-healing abilities to promote cell and tissue growth in vivo [5]. This is accomplished using tissue engineering methods in combination with other in vivo therapies such as cell or gene therapy, pharmaceutically optimized diets, or immunomodulation [3,4,6].

Tissue engineering and regenerative medicine (TERM) is the integration of medicine and bioengineering, which has resulted in the two having become widely interchangeable terms, as both fields focus on restoring tissue functionality to the body [3,6]. While TERM research has been conducted for decades now, its practice is still relatively new. It is a rapidly developing area of research that is being widely applied to nearly every specialty in medicine.

Tissue regeneration is performed by implanting cells and biomaterials into the body, which rebuilds tissues and supports its native self-healing abilities to promote tissue growth [7]. The biomaterials used often involve stem cell technology to promote cell growth [8]. By engineering tissue in vitro, one can create tissue mimics outside of the body to predict how the tissue growth would occur prior to implantation.

Tissue engineering holds a wide potential to increase the chance of successful outcomes in many organ systems in which the regeneration of damaged tissue is biologically unable to occur [7]. Tissue engineering can be used to regenerate our own tissue using our own cells [8,9,10,11,12]. For example, in cases of cell death involving cartilage or nerves, natural regeneration is unable to occur [2]. Therefore, surgical intervention or implantation of specialized stem cells is necessary for successful regeneration. Additionally, tissue defects can be genetic or acquired—due to birth defects, aging, accidents, or disease—and range from localized damage to whole organ failure [2,6,13,14]. The role of tissue engineering differs based on the extent of tissue damage. Here, we delve into the use of 3D bioprinting in specific preclinical models both in vitro and in vivo to further improve the field of TERM while also focusing on the specific materials and techniques that comprise 3D bioprinting.

## 2. Organ Transplantation

Organ transplantation has been a cornerstone of treatment for end-stage organ failure since the mid-1900s [8,15]. The first successful organ transplant occurred in 1954 when a kidney was successfully transplanted [16]. Since then, organ transplants have been widely used in clinical practice. However, as the demand for organ transplants has grown substantially over the decades, maintaining a constant supply of available organs has become difficult. According to the World Health Organization (WHO), it is estimated that only about 10% of those in need of an organ transplant receive one, while the number of patients waiting for an organ has constantly been growing over the years [17]. In 2019, according to the United Network for Organ Sharing (UNOS), the United States set an all-time high in the number of organ donors and transplants, with approximately 40,000 organ transplant surgeries [18,19]. Despite this significant milestone, it is estimated that more than 110,000 patients remained waiting for an organ transplant by the end of the year [18]. According to Health Resources and Services Administration (HRSA), around 17 people die every day while waiting for an organ [20]. These numbers are only increasing as more and more patients are added to the UNOS waiting list each year. For these reasons, there is an immense need to explore alternative organ sources.

## 3. Classical Approaches to Tissue Engineering

The most common approaches to tissue engineering include using (1) cells, such as for stem cell implantation, (2) bioactive molecules for the delivery of growth factors or other regulators, and (3) a combination of cells and biomaterials seeded into a porous 3D scaffold that can be implanted in vivo to promote natural cell growth [3,21,22,23]. It is important to gain a complete understanding of these traditional methods in order to identify optimal bioprinting applications.

### 3.1. Cells

**Figure 2:**
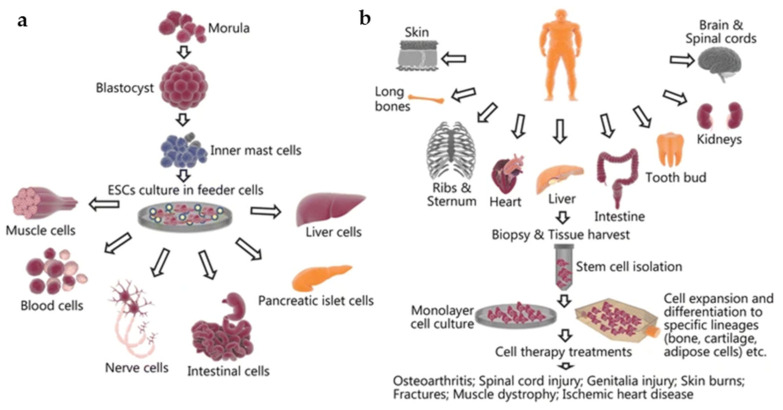
The harvesting and culturing process as well as potential tissue applications of (**a**) embryonic stem cells and (**b**) mesenchymal stem cells. Reproduced under open access from [25], published by BMC, 2018.

Many different cell types have been used in tissue engineering over the years. The classical applications of cell implantation involve either seeding scaffolds with cells and biomaterials in vitro or direct cell therapy via the injection of stem cells into the native tissue or organ of interest [24]. The selection of a specific cell type is arguably the most crucial step in tissue engineering, as it determines the functionality and design of the tissue-engineered model [12]. One of the most widely used cell types is stem cells [12]. Stem cells are able to differentiate into a specialized cell type of interest while also continuously dividing and renewing themselves throughout their lifespan [9,10,11,12]. This makes them attractive candidates for use in tissue engineering, and they have shown success in creating functional tissues that mimic the body’s natural organs. Stem cells can be isolated from different sources, including embryonic stem cells or mesenchymal (adult) stem cells. The choice of stem cells determines the differentiation fates of the cells [2,9]. For example, embryonic stem cells have the ability to differentiate into, but not limited to, blood or nerve cells (Figure 2a), while mesenchymal stem cells harvested from adult tissue have been found to differentiate into bone or cartilage cells, among others (Figure 2b) [25]. A recent study by Jo et al. found that mesenchymal stem cells have beneficial effects on skin regeneration by promoting cell proliferation, decreasing skin inflammation, and increasing collagen and elastic fibers [26]. Another study in rats found that the direct injection of mesenchymal cells in radiation-wound areas in the skin of rats resulted in an injury area of about half the size when compared with the control group of rats at 14 days post injection [27].

Clinically, autologous cell sources are preferred, meaning the cells are derived from the same individual that they will be used in, which decreases the risk of host rejection or other complications [24]. While allogenic (or donor) cells can be utilized in cases when autologous tissue is lacking or inadequate, the use of allogenic tissue can often result in difficulties due to the risk of a graft-versus-host reaction, unavailability of a matching donor, or the need for lifetime immunosuppression [24,28,29,30,31].

### 3.2. Bioactive Molecules

Bioactive molecules include various types of factors that can be integrated into the natural tissue. Some examples of bioactive molecules that are used in tissue engineering include growth factors and other signaling molecules, structural proteins, angiogenic factors, cytokines, hormones, DNA, RNA, or environmental stimuli [2,9,32,33,34,35]. The purpose of administering bioactive molecules is to enhance the host’s stem cell response to regenerate tissue more effectively [35]. According to Kim et al., bioactive molecules regulate cell movement, growth, and differentiation while also interacting with the transplanted cells or host cells in their specific microenvironments to promote tissue regeneration [32]. Bioactive molecules also impact functions such as influencing cell division and adhesion [2]. Overall, the incorporation of these bioactive molecules is crucial to successfully influence cell behavior and provide the nutrients needed to mimic the in vivo tissue environment [2].

The addition of growth factors is a common application of bioactive molecules. Growth factors play an important role in guiding stem cell differentiation, promoting cell growth, and acting as signaling molecules among cell populations, ensuring accurate tissue regeneration [34,35,36]. Additionally, while the scaffold provides the main physical support in tissue regeneration, the addition of structural adhesive proteins can further assist the tissue in developing proper mechanical properties [35]. For example, one study delivered bone morphogenetic protein-2, a transforming growth factor beta, via the implantation of a polyelectrolyte film, resulting in successful bone regeneration in rats [37]. However, various studies have reported difficulties in growth factor delivery due to their limited half-life, rapid degradation following delivery, and overall poor cost efficiency [38]. The use of chemotactic signals is another application in which bioactive signaling molecules trigger the recruitment of host stem cells to areas of tissue damage. Mobilization of stem cells to the impaired tissue site encourages natural regeneration and promotes cell proliferation and tissue repair [11]. Overall, further studies are needed to optimize the delivery of growth factors and bioactive molecules to promote tissue engineering, which further supports the need for alternatives to classical tissue engineering approaches.

### 3.3. Scaffolds

A scaffold consists of a physical replication of native tissue that is used in combination with stem cells and other biomaterials for optimized tissue engineering [2]. A biomaterial is defined as a “material intended to interface with biological systems to evaluate, treat, augment or replace any tissue, organ or function of the body,” making it an important factor in guiding TERM [14]. The functionality of scaffolds is intended to mirror that of the extracellular matrix in our bodies [23,39]. Firstly, the scaffold is designed to act as structural support to fill a tissue void in the area of interest [13]. Next, biomaterials or living cells can be seeded into the porous structure of the scaffold either as it is being made or after [13]. As the biomaterials embedded in the porous structure of the scaffold combine with the body cells upon implantation, they will have the capacity to promote stem cell differentiation and growth, ultimately allowing for the regeneration of native tissue [14].

The use of scaffolds has led to significant advances in tissue engineering in the past decade [23]. The conventional use of scaffolds in tissue engineering involves collecting stem cells from bone marrow, culturing and expanding these cells in vitro, and seeding them into biocompatible scaffolds [23,39]. This scaffolding construct then develops within a bioreactor, and the proliferation of the stem cells into their differentiated form is assessed via immunohistochemistry staining, scanning electron microscopy, or other cell assays [40,41]. Once the mature, specialized tissue of interest has formed properly, the tissue replica can be implanted in vivo [41]. Bioreactors are discussed in further detail in Section 5.3.1.

Bone tissue engineering (BTE) is a promising alternative to bone allografts or autografts through the idea of using cell-friendly scaffolds embedded with stem cells and biofactors to fill bony deficits within the body [42]. For example, one study prepared nano-hydroxyapatite/polyamide scaffolds embedded with mesenchymal stem cells [41]. These scaffolds matured over a period of 7 days, which was determined to be complete once the osteoblastic phenotype was identified. The scaffold was implanted in vivo in rabbit mandibles and found to promote natural bone formation and display excellent biocompatibility [41].

However, a major challenge with scaffolds in tissue engineering is the difficulty of neovascularization, which supplies blood flow and oxygenation to the tissue-engineered construct [43]. For this reason, in vitro tissue-engineered constructs must be limited in size and thickness. Upon in vivo implantation, angiogenesis could take several days, and perfusion will be unable to occur beyond a few hundred micrometers of the implanted tissue-engineered construct, which can cause damage to portions of the biofabricated tissue [43].

Overall, a more specialized, precise, and efficient alternative to these classical tissue engineering methods would have the potential to maximize the value of TERM. In recent years, 3D bioprinting has emerged as a viable alternative to these methods and seeks to address some of the challenges faced by classical tissue engineering methods [44].

### 3.4. Three-Dimensional (3D) Printing and Bioprinting

Three-dimensional (3D) printing uses computer-aided design (CAD) and segmentation software to sequentially layer 2D medical images (i.e.,: CT, MRI, etc.) into 3D models stored as digital files (i.e.,: STL, AMF) that can be printed into physical 3D structures [5,45,46]. The 3D printing technology is being utilized in many specialties of medicine for surgical planning, educational modeling, and the creation of implantable medical devices, etc. [15]. Conventional 3D printing uses a nonbiological, acellular material such as powders or gels to create the 3D printed object [15,44]. However, when a 3D printer uses biological, living cells (bioink) as the material to print the structure, this process is known as bioprinting [15].

Conventional 3D printing techniques and additive manufacturing have been used to print cell-free scaffolds for implantation in surgery, and 3D bioprinting is now being explored as a technology to assemble living cells, biomaterials, and biochemicals in functional tissue-like structures [44,47,48]. 3D bioprinting has evolved from the conventional process of first 3D printing scaffolds, followed by seeding them with cells to a simultaneous process that creates 3D-bioprinted matrix and cells concurrently [49]. Upon implantation of these cell-laden biological structures, 3D bioprinting has the potential to integrate the engineered tissue into the natural tissue, which will allow for restoration of natural tissue and organ function [8,48,50,51]. The potential clinical applications and examples of 3D bioprinting in tissue regeneration are presented in Section 6.

Additionally, cells are precisely layered by CAD and printed by 3D printers to exactly resemble the patient’s ECM, allowing for precision and customizability with 3D bioprinting [44,52]. Overall, it is important to explore 3D bioprinting as an alternative to conventional tissue engineering methods. The practice of 3D bioprinting has become increasingly popular in scientific research due to its ability to convert 3D imaging into 3D models embedded with living cells and active biomaterials, opening a new door in the realm of TERM applications.

## 4. Advantages of 3D Bioprinting

While conventional tissue engineering approaches have demonstrated success in the past, it is important to consider the limitations of reconstructing patients’ natural tissues through these methods. Some limitations of classical tissue engineering methods include inaccurate scaffold creation in comparison to the natural tissue’s anatomy, restrictions on biomaterials that can be delivered by classical engineering methods, or unreliable delivery of cells, and improper interactions between different cell lines upon implantation in vivo [53]. Additionally, some artificial in vitro structures can be incompatible upon application to different in vivo environments. This can cause undesirable interactions, risking increased cell damage at the area of interest [2,5,15,46]. Furthermore, while organ transplantations can provide beneficial results, the risk of graft-versus-host reactions and immunological complications can hinder successful outcomes in many cases [5,15,17].

There are many advantages of 3D bioprinting over conventional tissue engineering methods. Three-dimensional bioprinting enhances these older methods to implement a more automated process while also allowing for high precision and customization for every application [44]. Although scaffolds have already been well utilized in TERM over the years, they are limited in their ability to fully replicate the native extracellular matrix (ECM) of the body [1,9,14,49]. The utilization of 3D bioprinting in scaffold construction has made scaffolds’ microstructures more advanced and precise in their anatomical features, allowing for more accurate co-deposition of cells and biomaterials when compared with conventional tissue engineering methods [54]. Additionally, from a technical standpoint, the process of using a 3D bioprinter to create models based on medical images allows for the fabrication of complicated and complex biomimetic tissue systems [53]. The ability to make 3D-printed tissue replicas provides the engineer and physician more control over the spatiotemporal placement of cells and biomaterials due to the layer-by-layer construction [44]. This also allows for the customization of key anatomical features within the tissue replica like the interconnected pores and the sizing and placement of blood vessels, which can improve neovascularization, perfusion, and cellular communication while also allowing for larger 3D-bioprinted tissues to be created [43,55].

The wide range of biomaterials that can be used, along with the ability to customize bioinks plays a significant role in generating more realistic models to be placed into an in vivo setting for tissue regeneration [44]. Bioinks can be customized with specific growth factors or signaling molecules that can further expedite and improve tissue regeneration in vivo. For example, Lee et al. demonstrated that upon simultaneously embedding growth factors into bioprinted scaffolds, their release and delivery were enhanced by the 3D patterning of cells in the bioprinted scaffold [56]. Lastly, 3D bioprinting offers the ability to create tissue components from the patient’s own undifferentiated stem cells, which are immunotolerant because they are taken from the patient’s own bone or fat marrow. This reduces the risk of rejection upon in vivo implantation, thus avoiding the graft-versus-host reaction, a crucial advantage of 3D bioprinting [57,58]. Overall, using 3D bioprinting for tissue regeneration will result in increased accuracy with regard to native morphology, anatomy, porosity, and other features of the regenerated tissue.

## 5. Process of Bioprinting

**Figure 3:**
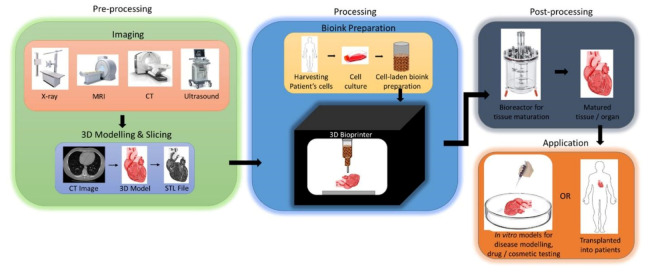
An overview schematic of the typical three-stage process of 3D bioprinting leading to the final in vitro or in vivo application. Reprinted with permission from ref. [44]. Copyright © 2018 Elsevier B.V.

The process of 3D bioprinting involves several important steps, which can be described as three important stages: (1) preprocessing—the creation of the digital 3D model to be printed; (2) processing—the creation of the bioink, and the actual process of bioprinting; (3) postprocessing—the stabilization, and maturation of the bioprinted 3D model [7,14,36,44,48,59] (Figure 3). Each of these stages includes several important steps that are crucial for the proper preparation of the materials used in bioprinting, the actual process of printing, as well as the maturation of the bioprinted model post-production.

### 5.1. Preprocessing

The preprocessing stage consists of detailed planning of the steps prior to the actual production of the bioprinted tissue [36]. This stage includes the two key steps of image acquisition and the digital creation of the 3D model.

#### 5.1.1. Image Acquisition

**Figure 4:**
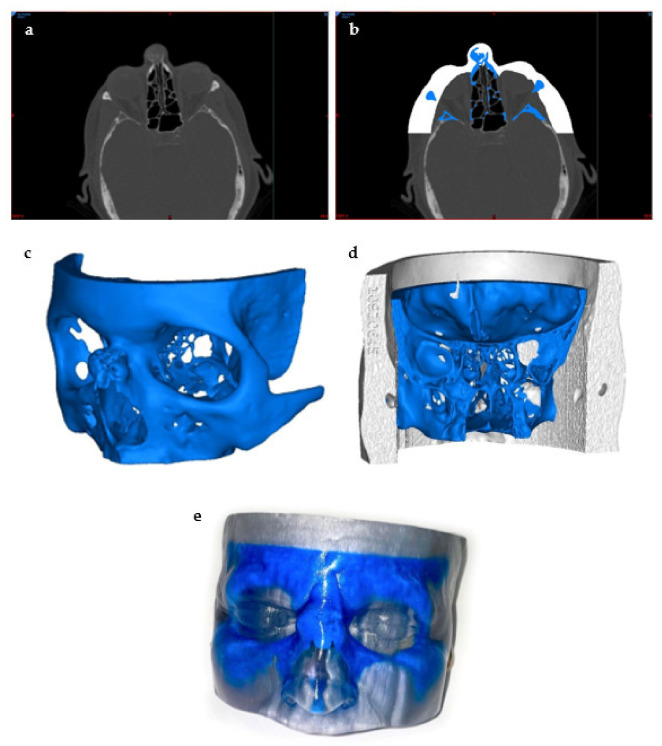
Preprocessing to 3D Printing of the 3D Model. This model was created to visualize a bone tumor in the nose: (**a**) visualization of the tumor using a CT scan of the head, (**b**) thresholding and segmentation to create separate masks of the skin and the tumor areas of interest using the segmentation software, (**c**) STL file creation of the bone tumor, (**d**) posterior view of the final STL model prior to printing, and (**e**) a photo of the final 3D-printed model.

Preceding the printing process, the first step of preprocessing is to image the tomography of the tissue of interest and gain an understanding of its basic anatomical properties. This is usually achieved using conventional 2D imaging methods such as MRI, CT, or ultrasound [59] (Figure 4a). Other imaging modalities used to visualize the tissue of interest include positron emission tomography (PET), single-photon emission computed tomography, or mammography [44,60,61]. The choice of imaging modality largely depends on the area of interest of the tissue or the characteristics of the tissue while also determining the resolution and accuracy of the 3D model to be created [62]. For example, MRI tends to favor the imaging of soft tissue, while CT is good for bones and other hard tissues [44]. In addition, a hybrid of imaging modalities can be used if needed, as shown by Kim et al., who used an overlap of CT and MRI images to image specific tissues [63].

#### 5.1.2. Designing the 3D Model

The second step of preprocessing is the designing of the 3D model using computer-aided design (CAD) software. This step is crucial in ensuring a high level of accuracy of the physical properties upon creating the 3D tissue mimic. There are many CAD software programs that are in use in medicine today, and the use of CAD software allows for increased efficiency by partially automating the design of the 3D structure in a way that follows the exact internal and external geometry while also ensuring low porosity of the structure in order to avoid future problems [64,65]. Firstly, the 2D images are segmented and split into different masks by anatomical region (Figure 4b). Once all of the necessary masks have been segmented, this file can be converted to a stereolithography file (STL) format, the typical file format accepted by most bioprinters, for 3D reconstructions by CAD software [36,44] (Figure 4c). The image layers are stacked to create a digital 3D structure through CAD that can be modified manually to confirm the presence of details, smooth out any imperfections, and correct any computer errors that may have been generated by the automated process [36]. The 3D model is rendered by the segmentation of volumetric units, also known as voxels, which are digitally put together to build the 3D mimic [45]. The size of the voxels can be adjusted to accommodate for fine details by smaller triangles making up the 3D structure, while larger voxels can be assembled more quickly at the cost of minute details [45]. Additionally, the correct internal anatomy and pore structure must also be verified manually to ensure proper cell proliferation and tissue growth upon implantation of the bioprinted material (Figure 4d). This step is integral to the rest of the bioprinting process because it will assemble the physical structure of the tissue mimic, which will act as the scaffold [47,66].

### 5.2. Processing

The processing step consists of the actual printing and manufacturing of the 3D model by selecting a printing method as well as the bioink, which includes both the biomaterials and the cell line [36,59,66]. The selection of proper bioink characteristics is crucial in encouraging the adhesion, proliferation, and functionality of the bioprinted tissue construct [67]. An understanding of the basic anatomical features and functionality of the tissue of interest is critical to guide the proper choice of the cell line, which will determine the rest of the process of bioprinting as well as potential limitations [66]. This includes considering the source of the cells, their ability to be applied in different environments, their maturation capabilities, and even the physical consistency of the bioink [66]. For example, the process of bioprinting skin is much simpler than bioprinting nerves due to the quick maturation rate of skin cells and their regenerative abilities; the delicacy of nerve cells requires a bioink that would preserve nerve cell functionality [44,66]. For these reasons, a strong understanding of these characteristics would make the planning and preparation of the bioink much more efficient.

#### 5.2.1. Methods of Bioprinting

**Figure 5:**
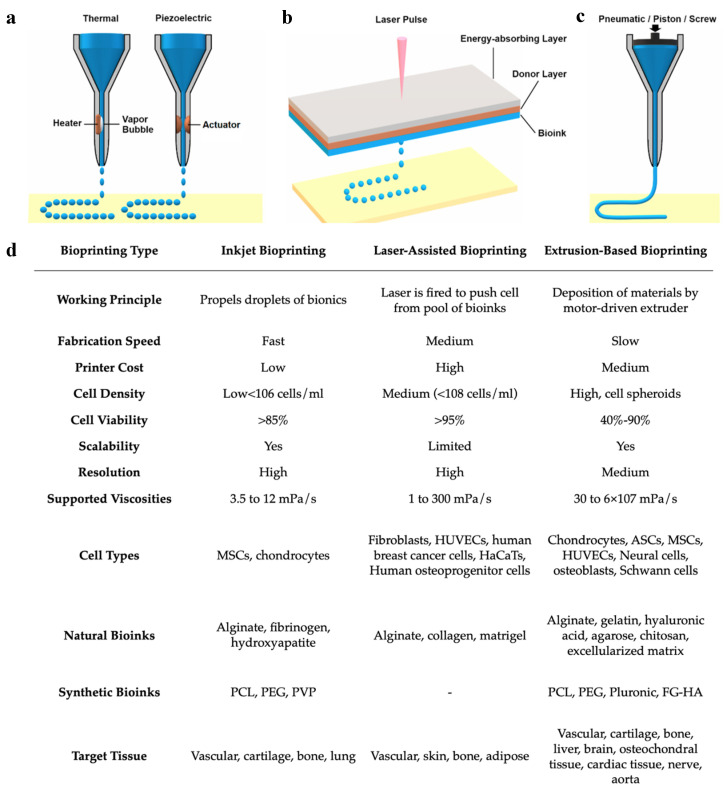
Comparison of (**a**) inkjet, (**b**) laser-assisted, and (**c**) extrusion-based bioprinting methodologies and their (**d**) working parameters. Reproduced under open access from [48] and [59], published by MDPI, 2020.

While bioprinting includes several different processes and methodologies, the three most commonly used bioprinting technologies include (1) inkjet bioprinting, (2) laser-assisted bioprinting, and (3) extrusion-based bioprinting (or pressure-assisted bioprinting) [52,59] (Figure 5a). Each of these methodologies has its own technical characteristics and determines the types of biomaterials that are compatible with the printer [59]. Some methodologies are favored over others to create certain tissues rather than others due to the type of bioinks that can be used. Inkjet bioprinting was derived from typical desktop printers, replacing the conventional ink cartridges with specialized bioinks to print living cells on a 3D structure [59]. Inkjet bioprinting functions as a non-contact printing process in which the liquid bioink is loaded into the nozzle and droplets are carefully deposited onto the surface of the tissue construct [36,68]. This process fabricates rapidly at a larger scale compared with other techniques [69]. Advantages of inkjet printing include the high resolution at about 50 μm, fast printing speeds, and low overall costs of production. However, the low viscosity of the bioink, which is required to avoid clogging the nozzle in inkjet bioprinting, weakens the structural integrity of the bioink and requires additional crosslinking to stabilize its structure [44,54,59,68]. Laser-assisted bioprinting uses monochromatic laser energy, either pulsed or continuous, to illuminate a ribbon-carrying bioink and a photoabsorbing layer, resulting in the creation of the 3D construct [52,70]. This process is non-contact and does not use a nozzle for the delivery of bioinks, resulting in high resolution, high cell viability, high cell densities, and fast production speeds [44,64,68,70,71]. However, disadvantages include high costs of maintenance, as well as the risk of cell damage caused by laser energy [44,64,70]. Depending on the laser source, laser-assisted bioprinting can be further classified as laser-induced forward transfer (LIFT), laser-guided direct writing (LG DW), matrix-assisted pulsed laser evaporation–direct writing (MAPLE DW), etc. [44]. Extrusion-based bioprinting is the most commonly used form of bioprinting and utilizes mechanical compressions or pneumatic pressure to continuously eject the bioink from the nozzle and deposit it in a layer-by-layer pattern [44,52,59]. The consistency of the bioinks used in extrusion-based bioprinting tends to be assembled as pastes or dispersions with higher viscosities compared to the other methods [52,54,72]. Due to the wide range of bioink viscosities, extrusion bioprinting is widely used to create large stable 3D tissue constructs [59]. In addition to the high viscosity of bioink that can be used, other advantages of extrusion-based bioprinting include low costs of production and high densities of cells that can be deposited [44]. However, disadvantages include slower production times and increased risk of the extrusion nozzle blockage by the bioink [44]. Most importantly, extrusion-based bioprinting has a very low resolution at about 100 μm [73]. It has also been thought that the stress of pressure forces may negatively impact cell viability and functionality [74].

Overall, each methodology has its own benefits and limitations. It is important to understand the key features of the tissue being reconstructed as well as the properties of each bioprinter before deciding on a bioprinting method (Figure 5b). As of now, no one technology has been isolated to display all the benefits at once. For example, laser-assisted bioprinting displays high resolution yet poor scalability, while extrusion-based bioprinting has low resolution and high scalability. The creation of a hybrid to combine the high resolution of inkjet bioprinting and the large scalability of extrusion-based bioprinting would be of great benefit in the field. Kim et al., used a hybrid of inkjet and extrusion bioprinting modules to create a 3D tissue construct of human skin [32]. They found the skin construct created to successfully promote cell growth while also being produced at reduced costs, requiring less material in the bioink [32]. These favorable results promote the use of hybrid bioprinting methods and encourage further research in this area.

#### 5.2.2. Creation of the Bioink

The production of the bioink is a challenging step in the bioprinting process, as it determines the overall functionality of the tissue construct [68]. Bioinks consist of two major components: the biomaterials and the cells [68,74]. Several considerations must be made upon choosing these components, including printability; the ability to withstand forces upon bioprinting; biocompatibility, i.e., the proper adherence and immune response of the cells; biodegradability, i.e., the ability to break down over time and allow for natural tissue regeneration to take over; mechanical characteristics, i.e., the correct anatomical features and structural integrity [14,59,70]. These factors must be accounted for upon the creation of the bioink.

Biomaterials act as support for the embedded cells by promoting adhesion, proliferation, and overall functionality of the growing cells [14,70]. There are three groups of biomaterials that are most commonly used in tissue engineering: natural polymers, synthetic polymers, and ceramics [14]. Natural polymers include biological materials that can be found naturally in the body, including compounds such as collagen, extracellular matrix, fibrin, silk, proteoglycans, etc. [14,68]. These are more commonly used in bioprinting due to their high biological activity and compatibility with native cells and microenvironments within the body [14,59]. They also promote degradation at a higher rate, allowing for the natural bodily reaction to take over and replace the implanted scaffold [14]. Natural polymers can also provide tissue-specific nutrients for the bioink [59]. Additionally, natural polymers can be reinforced by chemical or physical crosslinking to further strengthen polymers following bioprinting [75]. Synthetic polymers are another category of biomaterials that are chemically synthesized and incorporated into the bioink [59]. This group includes compounds such as polystyrene, polylactic acid, polyethylene glycol, polycaprolactone, and other manufactured polymers [14,59]. Although synthetic polymers are unable to degrade and replace extracellular matrices with the same efficiency as natural polymers, they have their own advantages, as they are highly specific and tailored towards the needs of the tissue construct [14,70]. This makes for the physical and chemical properties of the bioink, such as porosity or elasticity, to be easily manipulated and controlled [59]. Additionally, natural and synthetic polymers can be used in combination through a hybrid approach to improve the functionality of the bioink and produce a scaffold mimicking the natural ECM, including characteristics such as high biocompatibility, porous structure, mechanical stability, etc. [68,70,76,77].

While ceramics are not used as often as natural or synthetic polymers, this group is mainly used to recreate hard tissue constructs such as bones [14]. Ceramics consist of minerals such as calcium, phosphates, and hydroxyapatites, to name a few [14]. Scaffolds designed with a ceramic base tend to display stiff characteristics and low mobility, along with favorable interactions of osteogenic cells, which are all characteristics promoting bone structure [14]. Metals and polymers are also used as biomaterials in the fabrication of 3D-printed scaffolds. Metals exhibit strong mechanical stability, making them attractive candidates for making 3D-bioprinted bone tissue constructs, while polymers are used to create materials such as hydrogels, which display adjustable mechanical properties due to their hydrated nature and insolubility, allowing them to accurately mimic biological soft tissue [77]. Matai et al., provided a comprehensive summary of the materials used in various types of bioinks, as well as the functionality and success of the final biomimetic tissue [7].

#### 5.2.3. Choosing the Appropriate Cell Line

The proper selection of cells is believed to play a crucial role in tissue engineering, as previously mentioned. The chosen cell line determines the design and functionality of the tissue construct [12] (Figure 2). Together, the biomaterials and cells interact and determine the design of the tissue construct and the functionality of the bioink [12,59]. There are several factors to consider upon selection of the cells, including the cell source, intended function, number of cells, and cell viability [59,68,70]. Stem cells have an almost unlimited cell proliferation potential, making them the most commonly used primary cell line in tissue regeneration [11]. In addition to the primary cell line chosen, other cell lines can be incorporated to assist with the functionality and stability of the bioink [68]. For example, upon the bioprinting of blood vessels, pericytes can also be incorporated into the bioink to preserve the primary endothelial cells in the vasculature [68,78]. The number of cells and their delivery rates are key factors in tissue bioprinting. In order to create a smaller tissue construct, which calls for more details, single-cell dispersion allows for more control over details and precise delivery [70]. However, this method would not be preferred for the production of large tissues, in which cells can instead be dispersed in groups called spheroids [79]. Group delivery reduces production time and is also thought to provide improved cell viability. However, cell viability is influenced by the method of bioprinting used, crosslinking, and porosity [44,68,70] (Figure 5). Factors such as high printing speeds and nozzle pressures can decrease cell viability [71,80]. All final structural modifications are conducted by the bioengineer and are verified by the physician to ensure correct anatomical features before moving to the next stages. Once the bioinks and printing methods are finalized, the bioprinting of the tissue can occur.

### 5.3. Postprocessing

**Figure 6:**
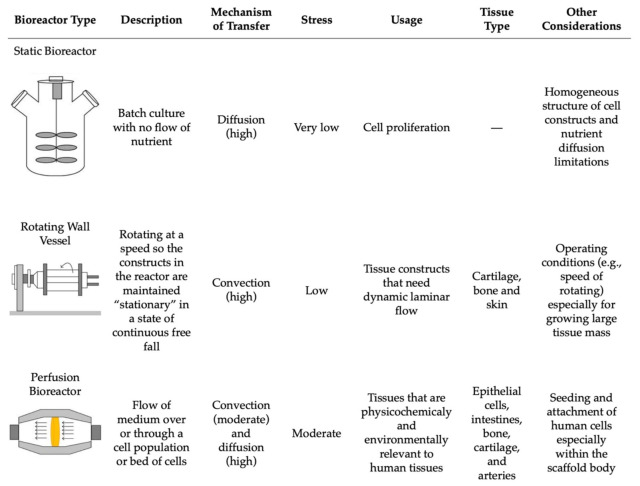
The comparison of the main bioreactors used in tissue engineering and their working parameters. Reproduced under open access from [84], published by Hindawi, 2013.

Lastly, the postprocessing step encompasses the growth and maturation of the bioprinted tissue [59]. This stage includes all of the steps after the 3D bioprinting is complete and before the in vivo implantation of the tissue construct [36]. Following production, the 3D tissue construct is held in in vitro conditions to mature before being implanted in vivo [68]. This step can be conducted in vitro through the use of bioreactors, where the environment mimics that of the natural tissue [59]. Bioreactors supply the new bioprinted tissue construct with nutrients and provide the chemical and physical stimulation needed for the differentiation and maturation of the cells [70,81]. There are various types of bioreactors that can be used based on the intended function, including static systems, perfusion bioreactors, and spinning/rotating vessels [81,82,83,84] (Figure 6). Within each of these systems, there are many variations and custom devices that have been designed to model the intricate physiological environment required to supplement the tissue with necessary nutrients. Some of these environmental cues include mechanical stimulation, fluid and compression stresses, gas exchange, and others to support tissue maturation [36]. The main difference between these three systems is their flow rate, resulting in differences in nutrient supply and stress transfer to the tissue construct [84] (Figure 6).

#### 5.3.1. Bioreactors

Static bioreactors are the simplest in terms of design and operational requirements. These systems work by incubating the cell culture in a static solution without any flow of nutrients, which can result in heterogeneously cultured cells [84]. This also results in the media having to be changed more often due to the buildup of wastes and excess nutrients [81]. Perfusion bioreactors tend to have a more complex structure and are commonly used for their homogenous mixing capabilities, allowing for the direct flow of nutrients through the tissue structure and resulting in better control of the cells [83]. Perfusion bioreactors are able to provide a continuous laminar flow of nutrients into the culture area while simultaneously removing any wastes [81]. Rotating wall vessels or spinning flasks are an alternative group of bioreactors that decrease the presence of gradients in the nutrients being delivered by applying more pressure to the tissue construct by the constant direct flow of nutrients [81]. However, similar to static systems, the media must be changed more often to replenish the nutrients for the cells [81].

Smith et al. created FABRICA, a 3D-printed bioreactor that cultures, perfuses, and analyzes the 3D bioprinted tissue construct [85]. Their study supported that the FABRICA bioreactor successfully perfused a 3D-bioprinted liver tissue, resulting in improved cell survival after one week when compared with a 3D-bioprinted liver tissue construct that was statically cultured for one week. Their data support the efficacy of bioreactors for 3D-bioprinted tissue while also demonstrating a need for advanced bioreactors to make progress in 3D bioprinting and tissue engineering [85]. Additionally, a bioprinted tissue construct can also be held in situ, for which the human body plays the role of the bioreactor and develops the tissue over time [59]. Overall, bioreactors allow for the bioprinted tissues to continue to develop into larger structures while adhering to the correct anatomy and functionality of the cells, allowing them to be precisely placed upon in vivo implantation [59].

## 6. Potential Clinical Applications of Tissue Regeneration

**Figure 7:**
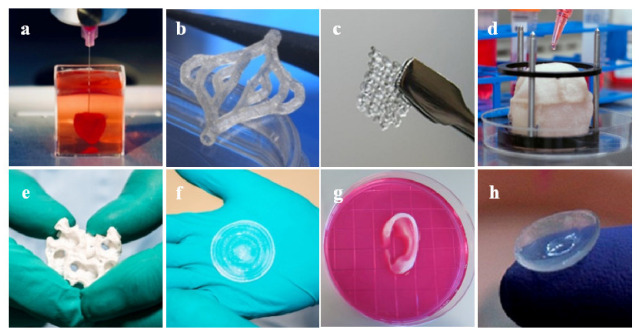
Examples of 3D bioprinted tissues: (**a**) heart [86], (**b**) blood vessels [87], (**c**) ovarian cells [88], (**d**) bladder [89], (**e**) bone [90], (**f**) skin [90], (**g**) ear [89], and (**h**) cornea [91].

There has been much progress in the realm of 3D bioprinting in the past decade, allowing for future applications within many areas of clinical medicine and, potentially, every major system in the body [44]. Due to the inability of certain tissues to regenerate naturally, surgical repair or artificial restoration are the mainstays of treatment [71]. Consequently, bioprinting has shown vast success in cases where organ transplant is difficult or not a viable option. Major body tissues such as the heart, blood vessels, and skin have seen success with 3D-bioprinted tissue implantation (Figure 7).

### 6.1. Cardiovascular

Hasan et al. developed a novel method to produce multi-layered blood vessels on a microfluidic device using a gelatin hydrogel. The investigators were able to create the physical structure of the vessels while ensuring the proper placement and growth of the endothelial cells within the vessel walls in three to five days of maturation [92]. Bertassoni et al. similarly saw success using agarose in a crosslinked hydrogel to form a printed blood vessel cultured with endothelial cells in vitro [93]. While direct implantation of bioprinted structures is one approach, others have investigated the use of bioprinting to accelerate the natural functions of the body. Gaebel et al. successfully bioprinted a cardiac patch cultured with mesenchymal stem cells and endothelial cells that was implanted on cardiac infarction zones in rats. The in vivo success of this preclinical study demonstrated the potential use of 3D bioprinting following a myocardial infarction to improve angiogenesis and assist in the recovery of the heart tissue [94].

### 6.2. Integumentary

Bioprinting can occur in situ as well by directly bioprinting onto the natural tissue. This has been observed in bioprinting for skin tissue, which is another organ system that has wide potential to help trauma or burn patients. Binder et al. directly implanted hydrogels made with keratinocytes and fibroblasts onto the skin of mice using a cartridge-based delivery system. They found successful wound healing and skin endothelialization eight weeks following implantation [95]. Additionally, many investigators have found success in bioprinting skin using traditional in vitro methods to create skin tissues [68,96,97,98,99,100,101,102,103]. Cubo et al. utilized in vitro methods to bioprint bilayer skin constructs derived from human plasma [99]. The skin patches were implanted on immunodeficient mice and demonstrated very similar characteristics to human skin upon maturation and contained all the functional layers of natural skin as well [99]. Follow-up studies have also been conducted to co-print other key anatomical features into the bioprinted skin such as sweat glands, hair follicles, and even melanocytes to regenerate fully functional tissue [104,105,106].

### 6.3. Musculoskeletal

Bone and cartilage are another set of tissues that have been successfully regenerated through bioprinting. Similar to the skin, there have been both in vitro and in situ methods proposed in bioprinting bones. Qi et al. used in vitro methods to prepare bioactive glass scaffolds consisting of calcium sulfate hydrate [107]. The authors found that the tissue construct exhibited complete adhesion and proliferation of the human mesenchymal stem cells while also enhancing the formation of natural bone tissue upon in vivo implantation in a rat model at an increased rate compared with the controls [107]. Regarding in situ applications, Keriquel et al. used laser-assisted bioprinting to create a bone construct using mesenchymal stromal cells, collagen, and hydroxyapatites to fill bone deficits in a mice model [108]. The authors found that the final tissue product was able to demonstrate complete functionality and viability compared with the controls, as well as showing proper osteoblast arrangement and proliferation capabilities [108].

Additionally, the bioprinting of cartilage has gained increasing importance over the years as cartilage is a tissue that cannot naturally be regenerated. For this reason, bioprinting is crucial to decrease the complications associated with cartilage degradation. Cui et al. used inkjet bioprinting to deposit a bioink consisting of chondrocytes and PEGDMA into a 3D biopaper plug and cultured it within a bioreactor for six weeks [8]. Following incubation, they found that the cartilage construct contained a lower amount of collagen I and a larger amount of collagen II when compared with a natural section of cartilage. This exhibits the proper maturation and growth of the cartilage cells over the incubation period [8].

## 7. Challenges, Future Directions, and Conclusions

The 3D bioprinting technology has established itself as a promising innovation in the realm of tissue regeneration and even has additional potential applications beyond tissue regeneration. Bioprinting is already being used in cancer research, drug development and delivery, prosthetics, and even clinician/patient education [7,109,110]. Although bioprinting has many advantages compared with conventional tissue engineering methods, challenges in implementation and utilization still exist. For example, 3D-bioprinted tissue constructs are not yet seen in human clinical settings in practice due to the poor mechanical properties and the lack of long-term data to support sufficient stability of the biofabricated tissue [54]. These challenges are also related to the types of cells and biomaterials chosen as well as the method of bioprinting utilized [54]. There are many limitations of bioinks and bioprinters that make it difficult to choose an ink that exhibits all of the desired characteristics of a particular application [7,48]. The method of bioprinting chosen must be compatible with the tissue being printed as well as the selected bioink [7]. Current bioprinting technologies must also be enhanced to increase printing speeds, resolution, and the scalability of the cells of the bioprinted structures [44,48]. A focus on improving these issues can result in a breakthrough for 3D bioprinting.

Additionally, the cost efficiency of 3D bioprinting must also be considered, particularly in regard to the high costs of 3D printers, cellular materials, and even computer software [46]. Some organizations have hired dedicated engineers to design and segment the 3D models due to the considerable amount of time and training needed to properly create 3D models. Overall, the costs of maintenance and expansion of bioprinting technologies make it challenging to readily bring 3D printing capabilities to clinics [111].

Furthermore, the size of 3D-printed tissues also remains a challenge. Currently, bioprinted tissues tend to be small and consist of a few cell types, resulting in limited functionality and scalability [46,70,71,111]. In addition, 3D printers are often limited in printing space, which results in limitations on the maximum size of 3D-printed tissues while also limiting the ability to create 3D-printed whole organs. Even assembling smaller 3D-printed tissue constructs into a larger model would result in errors during assembly [111]. In addition to size constraints, direct 3D bioprinting is often limited by the simulation characteristics of current materials, resulting in difficulty mimicking the natural tissue of the body and printing whole organs [111].

Recently, more novel techniques and strategies are being explored to advance 3D bioprinting. For example, a recent study created a novel ceramic-based bioink consisting of calcium phosphate and found they were able to 3D bioprint bone-like tissue that hardens within minutes after being placed in water [112]. Another study conducted by Zhang et al. constructed a pair of robots that work together to create a large 3D-printed tissue model using cement materials, and the group recently secured a patent for further studies [113]. While these groups are in the early stages of discovery, these novel ideas could strongly advance the field of 3D bioprinting [54]. Future advancements in 3D bioprinting and its technologies have wide potential within the field of tissue regeneration by allowing for more complex tissue manufacturing and improved medical applications.
